# Calcium Pyrophosphate Deposition Disease in the Achilles Tendon

**DOI:** 10.5334/jbsr.1987

**Published:** 2019-11-22

**Authors:** Kai-Yu Ho, Jing Nong Liang

**Affiliations:** 1 University of Nevada, Las Vegas, US

**Keywords:** sonography, calcium pyrophosphate deposition disease, Achilles tendon

## Abstract

**Teaching point:** The classic sonographic presentation of calcium pyrophosphate dihydrate crystal deposits in the Achilles tendon is reported.

## Case History

**Figure 1:**
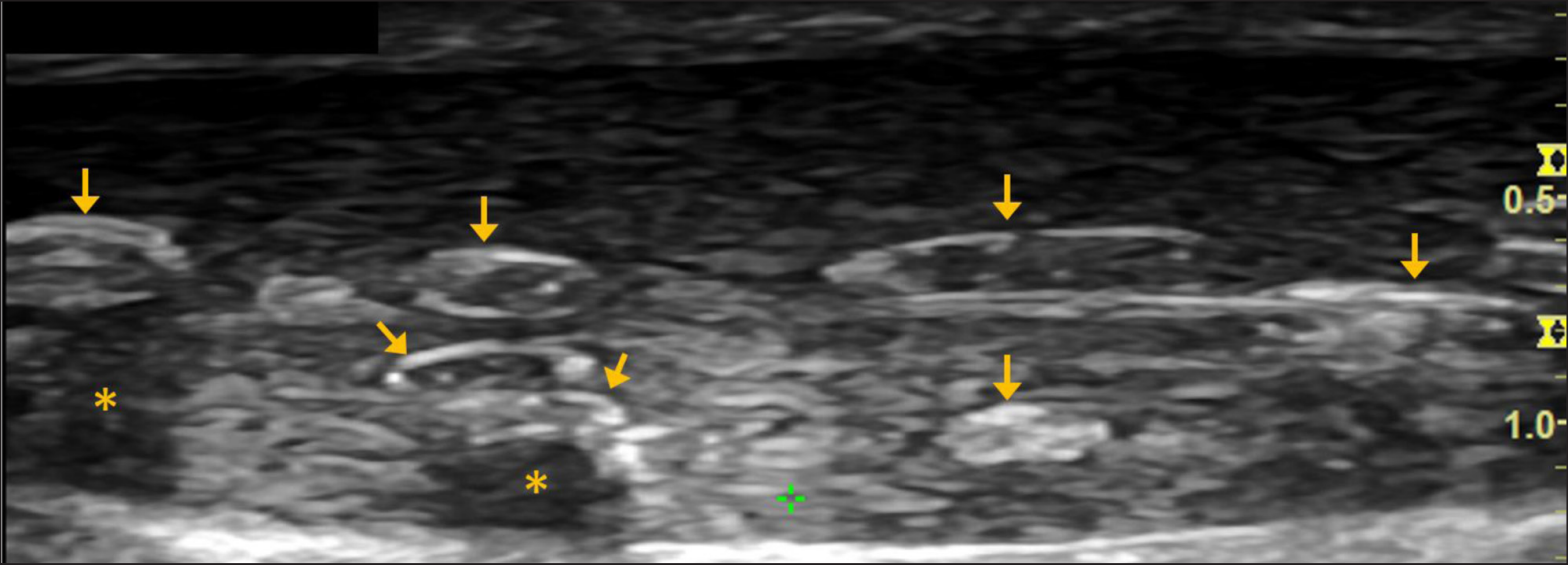


**Figure 2:**
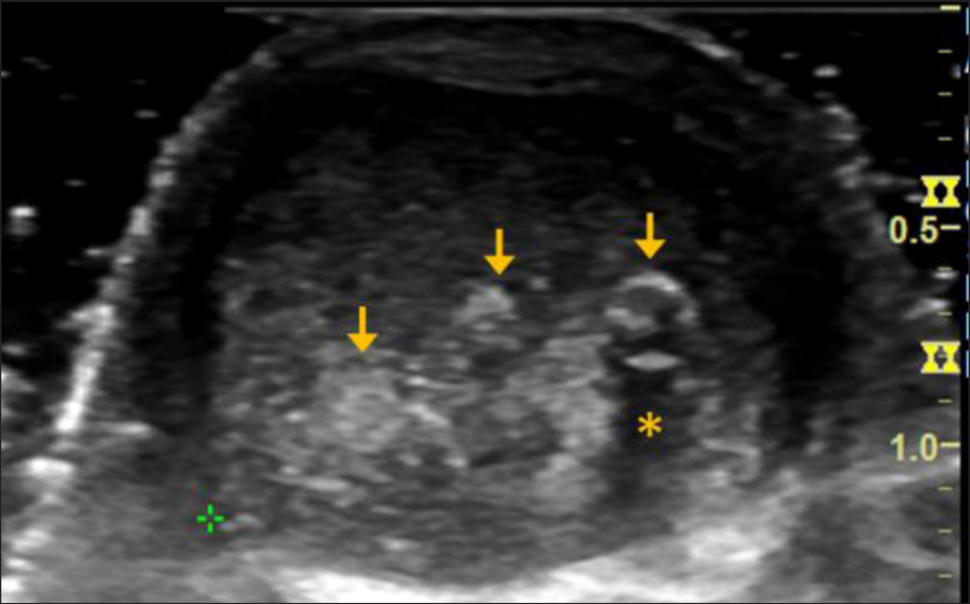


A 79-year-old male reported to physical therapy with occasional pain in the left Achilles tendon for over 20 years. He took pain medication on occasion to control symptoms but did not seek any medical advice prior to the visit. The sonography revealed multiple calcium pyrophosphate dihydrate crystal deposits in the longitudinal (Figure 1) and transverse (Figure 2) views of the left Achilles tendon. Specifically, calcium pyrophosphate dihydrate crystals were seen as characteristic linear hyperechoic bands positioned along the major axis of the tendon (arrows). The asterisks highlight the acoustic shadowing effect under the crystal deposits as a result of strong sound wave reflection from calcium deposition. The case was notified of the imaging findings and was followed up with an orthopedic physician for further examination and management on calcium pyrophosphate deposition disease (CPPD).

## Comment

In this report, we aimed to highlight the sonographic presentations of calcium pyrophosphate dihydrate crystal deposits in the Achilles tendon. Sonography has been suggested to be a good diagnostic tool for detecting calcium pyrophosphate deposition in the Achilles tendon with excellent sensitivity and specificity [1]. CPPD affects 4–7% of the adult population in Europe and the United States and often occurs at articular cartilage, fibrocartilage, and peri-articular soft tissues [1]. The Achilles tendon is a primary connective tissue affected by CPPD in the lower extremity [1]. While the underlying mechanisms leading to CPPD remain unclear, several risk factors/comorbidities have been reported, including older age (>60 years old), osteoarthritis, and metabolic conditions (e.g., hypophosphatasia, hyperparathyroidism, hemochromatosis, hypomagnesemia) [1]. The treatments for symptomatic CPPD primarily consist of rest, icing, and pharmacological therapies (e.g., Nonsteroidal anti-inflammatory drugs [NSAIDs] and glucocorticosteroid) to control pain and inflammation [1]. If CPPD is associated with an underlying condition (e.g., hyperparathyroidism), specific treatment targeting at the comorbidity would be necessary [1].

In conclusion, linear hyperechoic bands positioned along the major axis of the tendon from sonography refer to the presence of calcium pyrophosphate dihydrate crystals. The sonographic presentations of the Achilles tendon provided important insights into the management of the case’s clinical condition.
